# A dataset to measure global climate physical risk

**DOI:** 10.1016/j.dib.2024.110502

**Published:** 2024-05-04

**Authors:** Kun Guo, Qiang Ji, Dayong Zhang

**Affiliations:** a School of Economics and Management, University of Chinese Academy of Sciences, Beijing 100190, China; b Research Center on Fictitious Economy & Data Science, Chinese Academy of Sciences, Beijing 100190, China; c Institutes of Science and Development, Chinese Academy of Sciences, Beijing 100190, China; d School of Public Policy and Management, University of Chinese Academy of Sciences, Beijing 100049, China; e Research Institute of Economics and Management, Southwestern University of Finance and Economics, Sichuan 611130, China

**Keywords:** Climate risk, Extreme temperature, Extreme rainfall, Extreme drought

## Abstract

Extreme climate events have become more frequent and have had serious impacts on the global community. Consequently, the risk associated with climate change has gained increasing attention and has been considered as a new source of risk factors. To understand the socio-economic impacts of this new risk, systematically measuring risk around the world is critical for researchers and policymakers. Building on daily observations from meteorological stations, a Climate Physical Risk Index (CPRI) dataset is constructed for 170 countries, paying special attention to four extreme climate events: extreme low temperature (LTD), extreme high temperature (HTD), extreme rainfall (ERD), and extreme drought (EDD). A comprehensive index of climate physical risk for each country has also been constructed, covering the period from 1993 to 2023. The dataset will be updated regularly. Subnational indices or more detailed regional indices are available upon request.

Specifications Table

**Table:** 

Subject	Climate and Environmental Finance
Specific subject area	An interdisciplinary subject area connecting climate change and environmental issues with financing and investment issues.
Type of data	Table (Excel file)
Data collection	Our CRPI indices are constructed based on daily observations from meteorological stations worldwide. Four extreme climate events are considered and then combined to form a general index for each country. These events are extreme low temperature (LTD), extreme high temperature (HTD), extreme rainfall (ERD), and extreme drought (EDD). The first step is to define the “extreme” level. Following the literature, historical information, such as observations from 1973 to 1992, are used to determine the 10th and 90th percentiles of temperature, the 95th percentile of rainfall, and the 5th percentile of humidity, which are then used as thresholds. The total number of days in each of the four categories that exceed these thresholds in each year is calculated from 1993 to 2023. The four sub-indices are then standardized through a min-max approach (explained later). The mean value of these standardized sub-indices is used to represent the country specific CPRI.
Data source location	All raw data are downloaded from the National Oceanic and Atmospheric Administration (NOAA), Washington, DC, U.S.
Data accessibility	Repository name: figshare Data identification number: 10.6084/m9.figshare.25562229.v1 Direct URL to data: https://doi.org/10.6084/m9.figshare.25562229.v1

## 1 Value of the Data

• These data are useful in evaluating the degree of climate physical risk worldwide. Unlike some existing measures that use natural disasters and their associated economic impacts to construct such indices (e.g., Global Climate Risk Index | Germanwatch e.V.), the information here is more consistent, with better accuracy, in higher frequency, and can be extended to cover smaller regional climate conditions as the raw data is taken from actual observations by the meteorological stations at daily frequency.
• The core data covers information over 30 years for 170 countries and can be updated easily to provide a balanced panel. Given that the source is at the station level, our approach can be easily extended to calculate subnational or regional level of physical climate risks. Taking China as an example, the indices for 31 provinces and 229 prefectural cities are calculated.
• Although similar sources of information have been used by individual researchers, a comprehensive dataset open to the public and rich enough to capture different categories of climate events worldwide is yet to be available. This dataset can fill the gap by providing publicly available assessments of different types of climate physical risks.
• Researchers, business managers, financial analysts, and policy makers can use this dataset to analyze the macro or micro impacts of climate physical risk on the economy and society, thereby actively seeking strategies to mitigate the negative impacts of climate risk on macroeconomy, financial markets, firms and institutions, and others.

## 2 Background

Over recent decades, the frequency of extreme climate events such as heatwaves, extreme precipitation, and droughts has increased substantially [1]. Not only do these events cause direct losses to the global community through the destruction of infrastructure, reduction in productions, or increase in human mortality, but they also introduce significant uncertainties and indirectly lead to serious economic consequences, such as increasing systemic risks in the financial system [[2], [3], [4], [5]]. As a consequence, climate-related risks have drawn increasing attention in the literature and also been widely considered by practitioners and authorities. For example, the Basel Committee on Banking Supervision (BCBS) has issued principles for the effective management and supervision of climate-related financial risks.^1^

Typically, climate risks can be divided into physical risks and transitional risks [6]. While physical risk refers to the direct climate impacts and is often captured by extreme weather events or natural disasters [7], transitional risks are uncertainties due to efforts to mitigate climate changes, e.g., climate policy uncertainties [8].

In practice, meteorological data have been used to study climate impacts [9]. However, a general dataset covering climate physical risks worldwide has yet to be developed. Using a natural disaster database such as EM-DAT is one option [10], but it has notable problems compared to measurements build on meteorological information. The CPRI (Climate Physical Risk Index) dataset is constructed based on station-level meteorological observations. It can fill the gap and provide a flexible panel data easily adapted to different purpose of usage.

## 3 Data Description

The dataset stored in the repository [11] contains two Excel files, one referring to the global climate physical risk index (GCPRI) system for 170 countries, and another demonstrating the capability of extending the indices to sub-national or smaller regional level using the case of China, which includes Chinese climate physical risk indices for 31 provinces and 229 cities.

Each set of CPRI data contains four sub-indices and a total index, covering the period from 1993 to 2023. The contents of the repository are presented in Table 1. LTD (Extreme Low Temperature Days), HTD (Extreme High Temperature Days), ERD (Extreme Rainfall Days), EDD (Extreme Drought Days) are four sub-indices representing the number of extreme low temperature days, extreme high temperature days, extreme rainfall days, and extreme drought days in a country/region during a year, respectively. These indices are standardized using the method explained below and are then used to construct the general CPRI, which refers to the overall degree of climate physical risk of a country/region.

**Tabel 1:** Overview of the content of the repository.

File name	Sheet name	Sample	Indicators
Global Climate Physical Risk Index (GCPRI).xlsx	GCPRI	170 countries between 1993 and 2023	LTD HTD ERD EDD CPRI
Chinese Climate Physical Risk Index (CCPRI).xlsx	Province level	31 provinces of China between 1993 and 2023	LTD HTD ERD EDD CPRI
	City level	229 cities of China between 1993 and 2023	City LTD HTD ERD EDD CPRI

## 4 Experimental Design, Materials and Methods

### 4.1 Data collection

The raw meteorological data are sourced from NOAA (the National Oceanic and Atmospheric Administration).^2^ Daily climatological information from meteorological stations around the world (excluding special types of meteorological stations such as military bases and airports) from 1973 to 2023 is collected and used to construct the indices. The original indicators include daily average temperature, daily rainfall, and daily dew point observations. Here the dew point data is primarily used to calculate humidity in conjunction with the temperature data, which is then used to measure the risk of extreme drought. Historical data dating back to 1973 will be used to calculate the thresholds for extreme values. Specifically, 20 years of historical observations (from 1973 to 1992) are used for this purpose, therefore the effective CPRI data spans from 1993 to 2023.

### 4.2 Data preprocessing

**Step 1.** Missing value processing. If a meteorological station has a large amount of missing data, it is removed from the sample.

**Step 2.** Calculating the historical distributions of each indicator between January 1, 1973 and December 31, 1992. Define $T_{i}^{10}$ as the lower 10th percentile of the historical daily average temperature of station i, representing the threshold value for extreme low temperature; Define $T_{i}^{90}$ as the 90th percentile of the historical daily average temperature of station i, representing the threshold value for extreme high temperatures; $R_{i}^{95}$ is defined as the 95th percentile of historical daily rainfall of station i, representing the threshold value for extreme rainfall; $H_{i}^{5}$ is defined as the 5th percentile of historical daily humidity of station i, representing the threshold value for extreme drought.

**Step 3**. Counting the number of extreme days for each station from 1993 to 2023 for each type of event. The extreme low temperature days for station i in year n can be defined as $LTD_{i,n}$.

(1) $$LTD_{i,n}=\sum_{t=1}^{365}LT_{i,n,t}$$

(2) $$LT_{i,n,t}=\left\{ \begin{array}{c} 1\;if\;T_{i,n,t}<T_{i}^{10}\\ 0\;if\;T_{i,n,t}\geq T_{i}^{10} \end{array}\right.$$

Where $T_{i,n,t}$ represents the average temperature of station i in year n and day t. Similarly, extreme high temperature days $HTD_{i,n}$, extreme rainfall days $ERD_{i,n}$ and extreme drought days $EDD_{i,n}\;$can be calculated.

(3) $$HTD_{i,n}=\sum_{t=1}^{365}HT_{i,n,t}$$

(4) $$HT_{i,n,t}=\left\{ \begin{array}{c} 1\;if\;T_{i,n,t}>T_{i}^{90}\\ 0\;if\;T_{i,n,t}\leq T_{i}^{90} \end{array}\right.$$

(5) $$ERD_{i,n}=\sum_{t=1}^{365}ER_{i,n,t}$$

(6) $$ER_{i,n,t}=\left\{ \begin{array}{c} 1\;if\;R_{i,n,t}>R_{i}^{95}\\ 0\;if\;R_{i,n,t}\leq R_{i}^{95} \end{array}\right.$$

(7) $$EDD_{i,n}=\sum_{t=1}^{365}ED_{i,n,t}$$

(8) $$ED_{i,n,t}=\left\{ \begin{array}{c} 1\;if\;H_{i,n,t}<H_{i}^{5}\\ 0\;if\;H_{i,n,t}\geq H_{i}^{5} \end{array}\right.$$

Where $R_{i,n,t}$ and $H_{i,n,t}$ represent rainfall and relative humidity of station i in year n and day t, respectively.

**Step 4**. Calculating the annual number of extreme weather days at the regional level. Using the geographical coordinates of each station, the metrological data can be mapped to a certain region (country/province/city), so the average number of extreme days of the covered stations in the region can be calculated. Take the extreme low temperature days $LTD_{m,n}$ of the region m in year n for example, the formula is:

(9) $$LTD_{m,n}=\frac{1}{M}\sum_{j=1}^{M}LTD_{j,n}$$

Where M is the number of stations in the region, and $LTD_{m,n}$ is the arithmetic average extreme low temperature days of all stations in the area. Similarly, $HTD_{m,n}$, $ERD_{m,n}$ and $EED_{m,n}$ of the region m can be obtained.

### 4.3 Computation of the index

Given that the four individual extreme climate measures differ in nature, they are not directly comparable. The min-max standardization approach is used to process the data to construct a general index in each category. Taking the extreme low temperature days as an example:

(10) $${\bar{LTD}}_{m,n}=\frac{LTD_{m,n}-\min_{k=1,\cdots ,K;l=1\cdots ,L}\left\{LTD_{k,l}\right\}}{\max_{k=1,\cdots ,K;l=1\cdots ,L}\left\{LTD_{k,l}\right\}-\min_{k=1,\cdots ,K;l=1\cdots ,L}\left\{LTD_{k,l}\right\}}\times \;100$$

Where K is the total number of countries/regions, and L is the total number of years in the sample set. In this way, the four sub-indices LTD, HTD, ERD and EDD can be obtained.

After standardizing, the four sub-indices are used to construct a general climate physical risk index for each country/region. Here the simple average is used such as:

(11) $$CPRI_{m,n}=\omega_{1}{\bar{LTD}}_{m,n}+\omega_{2}{\bar{HTD}}_{m,n}+\omega_{3}{\bar{ERD}}_{m,n}+\omega_{4}{\bar{EDD}}_{m,n}$$

In this dataset, $\omega_{i}$ is set to 0.25. Obviously, the weights can be changed to serve any individual purpose in a particular project.

## Limitations

One of the main issues with this dataset is the use of simple average of the extreme climate days across all meteorological stations in a country/region, which essentially assumes that the importance of different meteorological station is the same. However, some stations may be located in the more central areas, whereas others may be located in sparsely populated areas. Of course, this concern is trivial for a global panel. For individual researchers focusing on a particular region, population density or night light data can be used to create a weighted average index to gain more accurate measures. Another problem may require attention is when extending to smaller regions, the availability of meteorological station data can be a limiting factor for the minimum size of the regional extension to be considered.

## Ethics Statement

The authors have read and followed the ethical requirements for publication in Data in Brief and confirmed that the current work does not involve human subjects, animal experiments, or any data collected from social media platforms.

## CRediT authorship contribution statement

**Kun Guo:** Conceptualization, Methodology, Software, Formal analysis, Visualization, Writing – original draft. **Qiang Ji:** Data curation, Investigation, Validation, Supervision, Project administration, Funding acquisition. **Dayong Zhang:** Investigation, Supervision, Validation, Writing – review & editing, Project administration.

## Data Availability

Climate Physical Risk Index (CPRI) (Original data) (figshare).

## Footnotes

1 https://www.bis.org/bcbs/publ/d532.htm.

2 https://www.ncei.noaa.gov/data/global-summary-of-the-day/archive/.
